# Biphasic (5–2%) oxygen concentration strategy significantly improves the usable blastocyst and cumulative live birth rates in in vitro fertilization

**DOI:** 10.1038/s41598-021-01782-6

**Published:** 2021-11-17

**Authors:** Sophie Brouillet, Chloé Baron, Fatima Barry, Aneta Andreeva, Delphine Haouzi, Anna Gala, Alice Ferrières-Hoa, Vanessa Loup, Tal Anahory, Noémie Ranisavljevic, Laura Gaspari, Samir Hamamah

**Affiliations:** 1 INSERM 1203, Développement Embryonnaire Fertilité Environnement, Univ Montpellier, Montpellier, France; 2 CHU Montpellier, Département de Biologie de la Reproduction, Biologie de la Reproduction/DPI et CECOS, Univ Montpellier, Montpellier, France; 3 CHU Montpellier, Unité d’Endocrinologie-Gynécologie Pédiatrique, Service de Pédiatrie, Univ Montpellier, Montpellier, France

**Keywords:** Biological techniques, Cell biology, Developmental biology, Molecular biology, Stem cells, Diseases, Health care, Medical research

## Abstract

Oxygen (O_2_) concentration is approximately 5% in the fallopian tube and 2% in the uterus in humans. A “back to nature” approach could increase in vitro fertilization (IVF) outcomes. This hypothesis was tested in this monocentric observational retrospective study that included 120 couples who underwent two IVF cycles between 2014 and 2019. Embryos were cultured at 5% from day 0 (D0) to D5/6 (monophasic O_2_ concentration strategy) in the first IVF cycle, and at 5% O_2_ from D0 to D3 and 2% O_2_ from D3 to D5/6 (biphasic O_2_ concentration strategy) in the second IVF cycle. The total and usable blastocyst rates (44.4% vs. 54.8%, *p* = 0.049 and 21.8% vs. 32.8%, *p* = 0.002, respectively) and the cumulative live birth rate (17.9% vs. 44.1%, *p* = 0.027) were significantly higher with the biphasic (5%-2%) O_2_ concentration strategy. Whole transcriptome analysis of blastocysts donated for research identified 707 RNAs that were differentially expressed in function of the O_2_ strategy (fold-change > 2, *p* value < 0.05). These genes are mainly involved in embryo development, DNA repair, embryonic stem cell pluripotency, and implantation potential. The biphasic (5–2%) O_2_ concentration strategy for preimplantation embryo culture could increase the “take home baby rate”, thus improving IVF cost-effectiveness and infertility management.

## Introduction

During in vitro fertilization (IVF), many exogenous factors can affect human embryo development, such as light, temperature, and chemical compounds^1^. One approach to increase IVF outcomes is to improve the in vitro microenvironment by mimicking the in vivo conditions, to promote embryo development and increase the implantation potential^1,2^. This “back to nature” approach concerns different parameters (e.g. temperature, pH, culture medium composition)^1,3^, including the gas composition inside the incubator during in vitro embryo culture^1,2,4^. Many studies provided evidence about the key role of oxygen (O_2_) concentration during preimplantation embryo development^2^. In the last 40 years, the majority of IVF laboratories worldwide used atmospheric (≈ 20%) O_2_ concentration for preimplantation embryo culture, mainly to limit the additional costs associated with the use of low O_2_ concentrations (e.g. requirement of a nitrogen gas system, specialized incubators, and additional quality assurance associated with oxygen sensors)^2,5,6^. However, a recent meta-analysis confirmed the significant increase in live birth rate when using monophasic 5% O_2_ compared with monophasic 20% O_2_^5^. Therefore, a continuous level of 5% O_2_ is currently used in most IVF laboratories for preimplantation human embryo culture^7^. Interestingly, recent data suggest that dynamic O_2_ exposure during in vitro culture might represent a more physiological environment, thus potentially improving IVF outcomes^8–12^. In humans, O_2_ concentration is approximately 5% in the fallopian tube from the fertilization stage to the cleaved embryo stage, and approximately 2% in the uterus from the morula to the blastocyst stage^8,13,14^. Hence, implementing a biphasic (5–2%) O_2_ concentration strategy in IVF laboratories seems relevant, especially because of the increasing use of extended culture up to the blastocyst stage^15^. Few studies reported a significant increase of the blastocyst rates in culture with biphasic (5–2%) O_2_ compared with monophasic (5%) O_2_ concentration^12,16^, but these results remain controversial^10^. Indeed, two studies found that the biphasic strategy led to an increase in the total blastocyst rate^12^ and the usable blastocyst rate^16^. Conversely, the third study reported similar blastocyst rates with both (monophasic and biphasic) strategies^10^. These discrepant results could be partly explained by differences in the women’s age among studies. Unfortunately, no information on maternal age was available in these three publications, which is a major limitation. Indeed, embryos generated using oocytes from younger women might display higher adaption to oxidative damage^17,18^, leading to similar blastulation rates when cultured in monophasic and biphasic O_2_ concentrations. In addition, the three studies used different definitions of usable blastulation (“ > 4CC”^12^, “≥ 1A with no C inner cell mass with concurrent C trophectoderm”^16^, and “from fully compacted embryos to hatching blastocysts with a visible trophectoderm and inner cell mass”^10^), although the 3BB threshold is usually considered as the minimal grade for blastocyst usability^19^. Moreover, they used different culture media that may include different antioxidants and free radical scavengers (no information available^12^, “one-step medium Global Total” from CooperSurgical®^16^, and “sequential medium” from Origio® ^10^). Furthermore, the molecular mechanisms by which the biphasic (5–2%) O_2_ concentration could improve in vitro embryo development and implantation potential are still unknown.

Hence, the aim of our clinical study was to evaluate the impact of biphasic (5–2%) O_2_ concentration on IVF outcomes compared with the monophasic (5%) O_2_ strategy during in vitro preimplantation embryo culture. The primary objective was to evaluate the impact of O_2_ concentration on human in vitro embryo development, with total and usable blastocyst rates as endpoints. The secondary objective was to characterize the impact of O_2_ concentration on clinical outcomes, with cumulative live birth rates (LBR) as endpoint. In addition, a transcriptomic study was performed to investigate the molecular impact of biphasic (5–2%) O_2_ culture on human embryo gene expression.

## Material and methods

### Ethics

The local research ethics committee approved the retrospective collection of clinical and biological data required for the clinical study (Institutional Review Board of Montpellier University Hospital, N° 2019_IRB-MTP_05-12). All patients included in the clinical study signed an informed consent for the collection of their clinical and biological data. The French Agence de la Biomédecine authorized the use of donated human IVF embryos for the in vitro study (NOR: SSAB1816140S). For the in vitro study, all involved patients signed an informed consent for the donation of cryopreserved embryos and for the collection of their clinical and biological data. Non-identifying study numbers were assigned to all data. All experiments were performed in accordance with the relevant guidelines and regulations.

### Clinical study design

**Figure 1:**
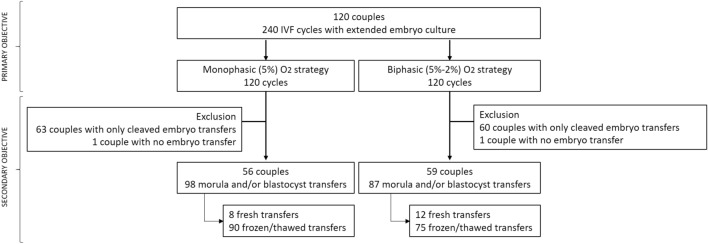
Study flowchart. IVF: in vitro fertilization.

This monocentric observational retrospective study was carried out at the Department of Reproductive Medicine of Montpellier University Hospital between June 2014 and March 2019. Inclusion criteria were couples undergoing IVF whose embryos were cultured in monophasic (5%) O_2_ concentration from day (D) 0 to D6 in one IVF cycle and in biphasic (5–2%) O_2_ concentration (i.e. 5% from D0 to D3, and then 2% from D3 to D6) in the subsequent IVF cycle. Exclusion criteria were: use of (1) testis or epididymal sperm^20–22^ (2) preimplantation genetic diagnosis, (3) donor oocytes, and (4) absence of growing embryos at D3. In total, 120 couples were included for evaluating the impact of O_2_ concentration on usable blastocyst rate (primary objective, Fig. 1). To assess the impact of O_2_ concentration on IVF outcomes (cumulative LBR; secondary objective), all cleaved embryo transfers were excluded. This left 98 embryo transfers from 56 couples after culture in monophasic O_2_ (5%), and 87 embryo transfers from 59 couples after culture in biphasic O_2_ (5–2%) concentration (Fig. 1).

### IVF procedures

#### Ovarian stimulation, ovarian puncture, and oocyte retrieval

Controlled ovarian hyperstimulation was obtained by pituitary downregulation with a Gonadotropin-Releasing Hormone (GnRH) agonist (Decapeptyl®) or antagonist (Orgalutran®, Cetrotide®) associated with follicle-stimulating hormone (FSH) (Gonal F®, Puregon®, Fostimon®) or a combination of FSH and luteinizing hormone (LH) (Pergoveris®, Menopur®, Fertistart®). Serum estradiol (E2) was quantified and follicle growth was evaluated (i.e. size and number of growing follicles) by transvaginal ultrasound from day 6 of stimulation. Circulating LH and progesterone levels were quantified to evaluate the risk of spontaneous ovulation. When at least three follicles reached a mean diameter ≥ 17 mm, recombinant human chorionic gonadotropin (hCG) (Ovitrelle®) or a GnRH agonist (Decapeptyl®) was administered for the final oocyte maturation. Ovarian puncture was performed 35 h after hCG/GnRH agonist injection by transvaginal ultrasound-guided needle aspiration of the follicles (Cook Medical Launches Otrieva™). This procedure was performed under conscious sedation, or occasionally under general anesthesia.

#### Gamete and embryo culture dishes

All culture dishes were prepared at room temperature the day before use, and equilibrated overnight in incubators (Heracell™ 240i, Thermo Scientific®) at a constant temperature of 37.0 ± 1 °C with 5% O_2_, 6% carbon dioxide (CO_2_) and 89% nitrogen (N_2_), or with 2% O_2_, 6% CO_2_ and 92% N_2_. Temperature, humidity, and gas content of the atmosphere inside the incubators were continuously controlled (external monitoring system Cobalt 2™, Oceasoft™).

#### Gamete collection and preparation

Cumulus-oocyte complexes (COCs) were retrieved from follicular fluid and placed in preincubated fertilization medium (G-IVF™ PLUS, Vitrolife®). They were cultured at 37 °C under a gas mixture of 5% O_2_, 6% CO_2_ and 89% N_2_ until insemination or denudation. For intracytoplasmic sperm injection (ICSI), oocytes were denuded using mechanical (Stripper, CooperSurgical®) and enzymatic methods (hyaluronidase: HYASE®, Vitrolife). Each semen sample was collected by masturbation in a sterile container after 2 days of abstinence. Sperm was prepared by density gradient centrifugation.

#### Gamete fertilization and embryo culture

All culture dishes (Micro-droplet Culture Dish®, Vitrolife) used from D0 to D5/6 were prepared at room temperature the day before use. After medium addition (G-IVF™ PLUS, G-1™ PLUS, G-2™ PLUS, OVOIL™, Vitrolife®), dishes were immediately placed without embryos in a CO_2_ incubator (HeracellTM 240i, Thermo Scientific®) overnight to equilibrate to 5% or 2% O_2_ at constant temperature (37.0 ± 0.2 °C).

Gamete fertilization and embryo culture were performed in pre-equilibrated culture embryo dishes at 5% O_2_, 6% CO_2_ and 89% N_2_ (HeracellTM 240i, Thermo Scientific®) from D0 to D3 (monophasic and biphasic strategies). In conventional IVF, the fertilization of each COC was performed in a pre-equilibrated culture dish (Micro-droplet Culture Dish, Vitrolife®) by adding 3000–3500 progressive motile spermatozoa in 20 µl droplets of fertilization medium (G-IVF™ PLUS, Vitrolife®) covered with mineral oil (OVOIL, Vitrolife®) at 38 h after oocyte maturation induction at 5% O_2_, 6% CO_2_ and 89% N_2_ (HeracellTM 240i, Thermo Scientific®). On D1, cumulus cells were mechanically removed (Stripper, CooperSurgical®) and each zygote was individually cultured in pre-equilibrated culture dishes (Micro-droplet Culture Dish, Vitrolife®) with 20 µl droplets of cleavage medium (G-1™ PLUS, Vitrolife) covered with mineral oil (OVOIL, Vitrolife®) at 5% O_2_, 6% CO_2_ and 89% N_2_ (HeracellTM 240i, Thermo Scientific®) until D3. For ICSI, spermatozoa were injected in denuded oocytes 39 h after oocyte induction trigger. Each injected oocyte was individually cultured in a 20 µl droplet of cleavage medium (G-1™ PLUS, Vitrolife) covered with mineral oil (OVOIL, Vitrolife®) at 5% O_2_, 6% CO_2_ and 89% N_2_ (HeracellTM 240i, Thermo Scientific®) in pre-equilibrated culture dishes (Micro-droplet Culture Dish®, Vitrolife) until D3.

Extended culture was performed from D3 to D5/6. Culture dishes (Micro-droplet Culture Dish®, Vitrolife) were prepared the day before use: 20 µl droplets of blastocyst medium (G-2™ PLUS, Vitrolife) were covered with mineral oil (OVOIL, Vitrolife®) and immediately placed without embryo in a CO_2_ incubator (HeracellTM 240i, Thermo Scientific®) overnight to equilibrate to 5% (monophasic strategy) or 2% O_2_ (biphasic strategy). At 70–73 h post- insemination/injection, developing cleaved embryos were placed individually in new pre-equilibrated dishes at 5% O_2_, 6% CO_2_ and 89% N_2_ (monophasic strategy) or at 2% O_2_, 6% CO_2_ and 92% N_2_ (biphasic strategy) up to D5/6 (HeracellTM 240i, Thermo Scientific®).

#### Embryo classification and selection

Each embryo was evaluated by two practitioners. Any embryo grading disagreement was resolved by discussion. The final decision was taken by a senior biologist. Embryo transfer and/or cryopreservation decisions were discussed and approved by a team of practitioners. Intra- and inter-observer quality controls were carried out in the laboratory by the internal and external quality referents several times per year. Normal fertilization was confirmed 17–20 h after insemination or injection by the presence of two pronuclei and the extrusion of the second polar body. Embryo growth was evaluated daily according to morphological and kinetic parameters. Embryo evaluation at D3 was based on the number and symmetry of blastomeres, the fragmentation percentage, the presence of multinucleated blastomeres, and the compaction degree. At D5/6, blastocyst morphology was evaluated according to the Gardner and Schoolcraft grading system^23^. Thus, in accordance with the literature, usable blastocysts were defined as full (grade 3), expanded (grade 4), partially hatched (grade 5), or fully hatched (grade 6) blastocysts with at least grade B trophectoderm quality^19^. Usable blastocysts were freshly transferred at D5 or cryopreserved at D5/6 for subsequent transfers. Early blastocysts (grade 1 or 2) at D5 were kept in culture until D6 and cryopreserved if considered as usable blastocysts at that point.

#### Embryo transfer and cryopreservation

The embryo transfer strategy was determined by a multidisciplinary team. Embryos were cryopreserved by vitrification and thawed following the manufacturer’s recommendations (Vit Kit-Freeze and Vit Kit-Thaw, FUJIFILM Irvine Scientific—BioCare Europe™). Frozen embryo transfers were performed on natural cycle, stimulated cycle or with hormonal replacement treatment. One or two embryos were transferred into the uterus using a catheter (Inventcath Eco®). Each woman received intravaginal progesterone for luteal phase support from the day of oocyte retrieval to the β-hCG blood test.

### Definition of clinical study endpoints

#### Primary objective

The total blastocyst rate was defined as the number of blastocysts (grade 1–6 according to the Gardner and Schoolcraft grading system^23^) at D5/6 divided by the total number of embryos in extended culture. The usable blastocyst rate was defined as the number of usable blastocysts at D5/6 divided by the total number of embryos in extended culture.

#### Secondary objective

For this objective, only the clinical outcomes of IVF cycles associated with fresh and frozen transfer of morulae (D4) and blastocysts (D5/6) were included. Endpoints were defined using the International glossary on infertility and fertility care^24^ and the cumulative rates proposed by^25,26^. The cumulative LBR was defined as the first live birth (i.e. a delivery associated with at least one live baby at > 24 weeks of gestation) obtained after transfer of fresh and/or frozen-thawed embryos derived from a single ovum pick-up^26^. The number of live newborns per cycle was defined as the total number of live babies at > 24 weeks of gestation obtained after transfer of fresh and/or frozen-thawed embryos derived from a single ovum pick-up. A completed cycle was defined as a cycle where all cryopreserved embryos had been thawed.

### Transcriptomic study design

As the impact of O_2_ concentration on embryo development is highly species-specific, data from animal studies can hardly be extrapolated to humans^27^. Therefore, a transcriptomic study was performed with human IVF cryopreserved blastocysts donated for research (n = 12) by eight couples. Gametes were collected, fertilized, cultured until the blastocyst stage, and cryopreserved in the laboratory following the same parameters (media, equipment, and protocols) and during the same period as the clinical study (2014–2019). All cryopreserved and thawed blastocysts (vitrification with the Vit Kit-freeze and thawing with Vit Kit-Warm, FUJIFILM Irvine Scientific) had high morphokinetic parameters. Patient data were collected from the patients’ clinical records.

#### Embryo transcriptome analysis

For each embryo, total RNA was extracted and purified using the Qiagen RNeasy Micro Kit (Cat#74004, Qiagen, Courtaboeuf, France) following the manufacturer’s instructions. RNA was eluted in 10 µl RNase-free water and stored at − 80 °C until analysis. Whole transcriptome analysis was performed using the Clariom D Pico Assay (Cat#902924, Thermofisher Scientific, Courtaboeuf, France) that allows assessing the expression of more than 540,000 coding and non-coding transcripts, including mRNA, circRNA, lncRNA, miRNA precursors and other small RNAs, using a small amount of total RNA^28,29^. Briefly, the Genechip Pico Reagent Kit protocol was first used to prepare hybridization-ready targets from picogram to nanogram quantities of total RNA samples. Total RNA was reverse transcribed to single-stranded cDNA containing the T7 promoter sequence at the 5’ end. Double-stranded DNA was synthetized in an in-vitro-transcription (IVT) amplification reaction by adding a 3’ adaptor as template. Double-stranded DNA was then used as template for antisense RNA synthesis and overnight amplification by IVT, using T7 RNA polymerase. Purified cRNA was then used for sense single-stranded cDNA (ss-cDNA) synthesis, followed by RNase H digestion and ss-cDNA magnetic bead purification. Ss-cDNA was fragmented using uracil DNA glycosylase and apurinic/apyrimidinic endonuclease 1, and then labeled by terminal deoxynucleotidyl transferase (TdT) using a proprietary DNA Labeling Reagent that is covalently linked to biotin. Finally, the hybridization cocktail was loaded into single human Clariom D arrays and incubated in the Affymetrix GeneChip Hybridization Oven 645 at 45 °C, 60 rpm, for 16 h. Arrays were stained using an Affymetrix GeneChip Fluidics Station 450, according to the specific fluidics protocol (FS450_0001), and scanned with an Affymetrix GeneChip Scanner 3000 7G. Raw intensity CEL files generated by GeneChipTMcommand ConsoleTM were imported into the Transcriptome Analysis Console (TAC) 4.0 (Applied Biosystems).

### Statistical analysis

Data were reported as mean and standard deviation for quantitative data and number/percentage for qualitative data. Data normality was assessed first by visual inspection of their distribution and then with the Shapiro–Wilk test. The non-parametric t test (Mann–Whitney) was used for continuous data, and the Chi-square and Fisher’s exact tests for categorical data. A *p* value < 0.05 was considered significant. Statistical analyses were performed using GraphPad Prism (GraphPad Prism 5.0, GraphPad Software Inc).

Microarray data were normalized and differentially expressed coding and non-coding RNAs were identified using the Transcriptome Analysis Console (TAC) software, with the following settings: Analysis Type: Expression Gene; Summarization Method: Gene Level—RMA; Gene-Level *P* value < 0.05; ANOVA Method: ebayes. All genes with significant expression changes (*p* < 0.05), and those with at least a two-fold change were selected.

The genetic network was generated with *Ingenuity Pathways Analysis* (IPA; Ingenuity Systems, www.ingenuity.com). The list of differentially expressed genes was overlaid onto a global molecular network developed based on the information contained in the Ingenuity Knowledge Base.

## Results

### Clinical study

#### Primary objective

The characteristics of the couples and the biological parameters are presented in Table 1. As expected, the women’s age (35.3 ± 4.4 years vs. 33.5 ± 4.6 years, *p* < 0.01) and total number of IVF cycles (2.7 ± 1.0 vs. 1.6 ± 0.9, *p* < 0.01) were significantly higher in the biphasic (5–2%) O_2_ strategy compared with the monophasic (5%) oxygen strategy. Moreover, the total blastocyst rate was significantly higher in the biphasic (5–2%) O_2_ than in the monophasic (5%) oxygen strategy (44.4% (230/518) vs. 54.8% (297/542), *p* = 0.049), as well as the usable blastocyst rate (21.8% (113/518) vs. 32.8% (178/542), *p* = 0.002) and the usable blastocyst rate at D5 (9.1% (47/518) vs. 19.9% (108/542), *p* = 1.53 × 10^–5^). All the other parameters were similar between groups.

**Table 1:** Characteristics of the couples and biological parameters of the IVF cycles used for the primary objective. Data are reported as means and standard deviations (SD). *BMI* body mass index, *IVF* in vitro fertilization, *IU* International Unit, *PN* pronuclei.

Parameter	Monophasic (5%) O_2_ strategy	Biphasic (5–2%) O_2_ strategy	*p* value
Couples (n)/cycles (n)	120/120	120/120	–
Women’s age (years)	33.5 ± 4.6	35.3 ± 4.4	< 0.01
Women’s BMI (kg/m^2^)	23.7 ± 4.3	23.8 ± 4.2	0.91
Men’s age (years)	36.4 ± 6.1	37.9 ± 6.1	0.06
Duration of infertility (months)	44.3 ± 29.1	43.7 ± 29.4	0.86
Total number of IVF cycles (n)	1.6 ± 0.9	2.7 ± 1.0	< 0.01
Number of women smoking (cigarettes/week)	25/120 50 ± 35	24/120 50 ± 35	1 1
Number of men smoking (cigarettes/week)	42/120 72 ± 39	42/120 75 ± 40	1 0.77
Primary dose of gonadotrophins (IU)	215.2 ± 82.9	234.2 ± 82.2	0.06
Total dose of gonadotrophins (IU)	2274.5 ± 979.1	2559.6 ± 1077.3	0.06
Duration of stimulation (days)	10.4 ± 1.5	10.8 ± 1.9	0.06
Retrieved oocytes (n)	10.9 ± 5.0	11.1 ± 6.0	0.86
Oocytes (n) inseminated or injected (IVF/ICSI)	247/843	219/860	0.18
2 PN embryos at day 1 (n)	5.7 ± 3.3	5.6 ± 3.4	0.52
Embryos at day 3 (n)	6.4 ± 3.7	6.2 ± 3.8	0.49
**Total blastocyst rate (n, %)**	**44.4% (230/518)**	**54.8% (297/542)**	**0.049**
**Usable blastocyst rate (n, %)**	**21.8% (113/518)**	**32.8% (178/542)**	**0.002**
**Usable blastocyst rate at day 5 (n, %)**	**9.1% (47/518)**	**19.9% (108/542)**	**1.53 × 10**^**–5**^
Usable blastocyst rate at day 6 (n, %)	12.7% (66/518)	12.9% (70/542)	0.94

Data in bold indicate statistically significant results.

#### Secondary objective

The cumulative LBR was significantly higher with the biphasic (5%-2%) than monophasic (5%) O_2_ concentration strategy (10/56 (17.9%) vs. 26/59 (44.1%), *p* = 0.027) (Table 2).

**Table 2:** Clinical and biological parameters of IVF cycles used for the secondary objective (i.e. cycles associated with morula or blastocyst transfers). *BMI* body mass index, *IVF* in vitro fertilization, *IU* International Unit, *PN* pronuclei.

Parameter	Monophasic (5%) O_2_ strategy	Biphasic (5–2%) O_2_ strategy	*p* value
Couples/embryo transfer cycles (n)	56/98	59/87	–
Mean number of embryo transfers per patient (mean ± SD)	1.75 ± 1.01	1.5 ± 0.69	0.22
Mean number of transferred embryos (mean ± SD)	1.2 ± 0.4	1.1 ± 0.3	0.33
Fresh/frozen-thawed embryo transfers (n)	8/90	12/75	0.22
Endometrial thickness in mm (mean ± SD)	9.33 ± 1.93	8.89 ± 1.63	0.43
**Cumulative live birth rate (n, %)**	**10/56 (17.9%)**	**26/59 (44.1%)**	**0.027**
**Number of live newborns per cycle n, % [min–max] per couple**	**10/56 (17.9%) [0–1]**	**30/59 (50.8%) [0–2]**	**0.009**
**Number of embryo transfers associated with at least one live birth (n, %)**	**10/98 (10.2%)**	**27/87 (31.0%)**	**0.004**
Gestational age of newborns in weeks (mean ± SD)	37.0 ± 2.0	35.7 ± 2.8	0.12
Birth weight of newborns in grams (mean ± SD)	3469 ± 527	3192 ± 578	0.11
Height of newborns in cm (mean ± SD)	51.0 ± 1.7	49.5 ± 2.1	0.35
Sex of newborns (male/female)	7/3	16/14	0.36
Completed cycles (all cryopreserved embryos used) (n, %)	56/56 (100%)	44/59 (74.6%)	0.29
Number of unused cryopreserved embryos (nb of couples)	0 (0)	38 (15)	–

Data in bold indicate statistically significant results.

Similarly, the number of live newborns per cycle (10/56 (17.9%) vs. 30/59 (50.8%), *p* = 0.009) and the number of transfers associated with at least one live birth (10/98 (10.2%) vs. 27/87 (31.0%), *p* = 0.004) were significantly higher with the biphasic (5%-2%) than monophasic (5%) O_2_ concentration strategy (Table 2). All the other parameters were similar between groups, including gestational age, birth weight and height, and sex of newborns (Table 2). At the study end, there were still 38 unused cryopreserved usable blastocysts to transfer from 15 couples in the biphasic (5%-2%) O_2_ group (versus 0 in the monophasic group) (Table 2). All of these 15 couples had at least one live birth.

### Embryo transcriptome analysis

For the transcriptome study, twelve blastocysts from eight IVF couples were analyzed: six blastocysts were cultured in monophasic (5%) O_2_ and six in biphasic (5–2%) O_2_ condition before cryopreservation. Table 3 shows the characteristics of patients and blastocysts.

**Table 3:** Clinical and biological parameters of the embryos donated for the transcriptomic analysis.

Parameter	Monophasic (5%) O_2_ strategy	Biphasic (5%-2%) O_2_ strategy	p value
Blastocysts (n)	6	6	–
Women’s age (years)	37.9 ± 2.9	36.2 ± 2.2	0.39
Women’s BMI (kg/m2)	21.7 ± 2.1	23.2 ± 2.0	0.39
Men’s age (years)	37.9 ± 4.0	42.1 ± 1.7	0.05
Anti-Müllerian hormone (ng/ml)	6.3 ± 6.0	4.4 ± 1.7	0.64
Antral follicle count	18.0 ± 8.8	14.6 ± 1.3	0.22
Primary dose of gonadotrophins (IU)	202.4 ± 73.3	160.0 ± 22.4	0.39
Total dose of gonadotrophins (IU)	1883.4 ± 595.6	2040.0 ± 22.4	0.22
Duration of stimulation (days)	9.8 ± 0.8	11.6 ± 0.9	0.05
Vitrification day (day 5/day 6)	5/1	4/2	1
Blastocyst morphology (B3/B4/B5)	3/1/2	3/1/2	1

**Figure 2:**
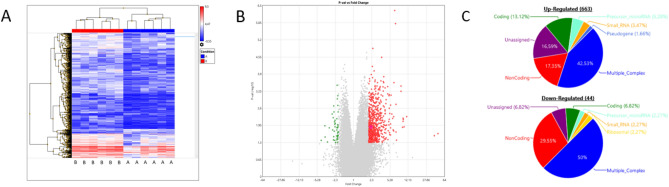
Transcriptome analysis of human embryos cultured in monophasic (5%) O_2_ or biphasic (5%-2%) O_2_ concentration. (**A**) Heat map. Group A (right part) includes six blastocysts cultured in monophasic (5%) O_2_ concentration; Group B (left part) includes six blastocysts cultured in biphasic (5–2%) O_2_ concentration. Upregulated genes are in red and downregulated genes are in blue. (**B**) Volcano plot. Each point represents one gene. On the X-axis, the fold change was calculated as the log2-ratio between the mean gene expression in embryos cultured in biphasic (5–2%) O_2_ concentration and the mean gene expression in embryos cultured in monophasic (5%) O_2_ concentration. Positive and negative values indicate the expression of genes which is higher in embryos cultured in biphasic (5–2%) and monophasic (5%) O_2_ concentration, respectively. On the Y-axis, the statistical significance of the difference in gene expression between culture conditions is represented as the (− log10) of the *P* value. Red and green points mark differentially expressed genes. The thresholds of significance are − 2 and + 2 for downregulated and upregulated genes respectively (x axis), and 1.30 (equal to a *P* value = 0.05) for the y axis. (**C**) Pie chart of the upregulated (n = 663) and downregulated (n = 44) transcripts in human IVF blastocysts cultured in biphasic (5–2%) O_2_ concentration compared with monophasic (5%) O_2_ concentration.

The transcriptome analysis yielded a mean of 135,750 transcripts per embryo (Fig. 2).

### Identification of differentially expressed genes in preimplantation embryos

The whole transcriptome analysis identified 707 RNAs that were differentially expressed in blastocysts depending on the O_2_ strategy (Fig. 2, fold-change > 2, *p* value < 0.05). Most of these RNAs were upregulated (663/707; 93.8%; red dots in Fig. 2B) in blastocysts cultured in biphasic (5–2%) O_2_ concentration compared with those cultured in monophasic (5%) O_2_ concentration. Conversely, 44/707 (6.2%) RNAs were downregulated in blastocysts cultured in biphasic (5–2%) O_2_ conditions. Multiple complex RNAs represented approximately 50% of the differentially expressed RNAs (Fig. 2C). Coding and non-coding RNAs represented 13.12% and 17.3% of upregulated RNAs, respectively (Fig. 2C). Coding and non-coding RNAs represented 6.82% and 29.5% of downregulated RNAs, respectively (Fig. 2C). The top-five upregulated genes were *VN1R84P* (vomeronasal 1 receptor 84 pseudogene, × 8,sevenfold, *p* = 0.0004, Multiple Complex), *SNORD14E* (small nucleolar RNA, C/D box 14E, × 6.55-fold, *p* = 4.92 × 10^–7^, Small RNA), *COX7B2* (cytochrome c oxidase subunit VIIb2, × 6.29-fold, *p* = 0.0185, Coding), *C3orf14* (chromosome 3 open reading frame 14, ×5.98-fold, *p* = 0.0062, Multiple Complex), and *DCPS* (decapping enzyme, scavenger, ×5.74-fold, *p* = 0.0042, Multiple Complex) (Table S1A). The expression of two hypoxia-induced genes [i.e. *HIGD2A* (HIG1 hypoxia inducible domain family member 2A, ×2.9-fold, *p* = 0.0067, Multiple Complex) and *HILPDA* (hypoxia inducible lipid droplet–associated), ×2.2-fold, *p* = 0.0131, Multiple Complex)] was significantly higher in blastocysts cultured in biphasic (5–2%) O_2_ than in monophasic (5%) O_2_ concentration (Table S1B). The top-five downregulated genes were *FKBP9* (FK506 binding protein 9, x-4.4-fold, *p* = 0.0413, Multiple Complex), *NEO1* (neogenin 1, x-3.7-fold, *p* = 0.0116, Multiple Complex), *TOM1L1* (target of myb1 like 1 membrane trafficking protein, x-3.04-fold, *p* = 0.0141, Multiple Complex), *LTBP1* (latent transforming growth factor beta binding protein 1, x-2.67-fold, *p* = 0.0042, Multiple Complex), and *KIAA0040* (KIAA0040, x-2.58-fold, *p* = 0.0166, Multiple Complex) (Table S1C).

### Functional annotation of differentially expressed genes

**Figure 3:**
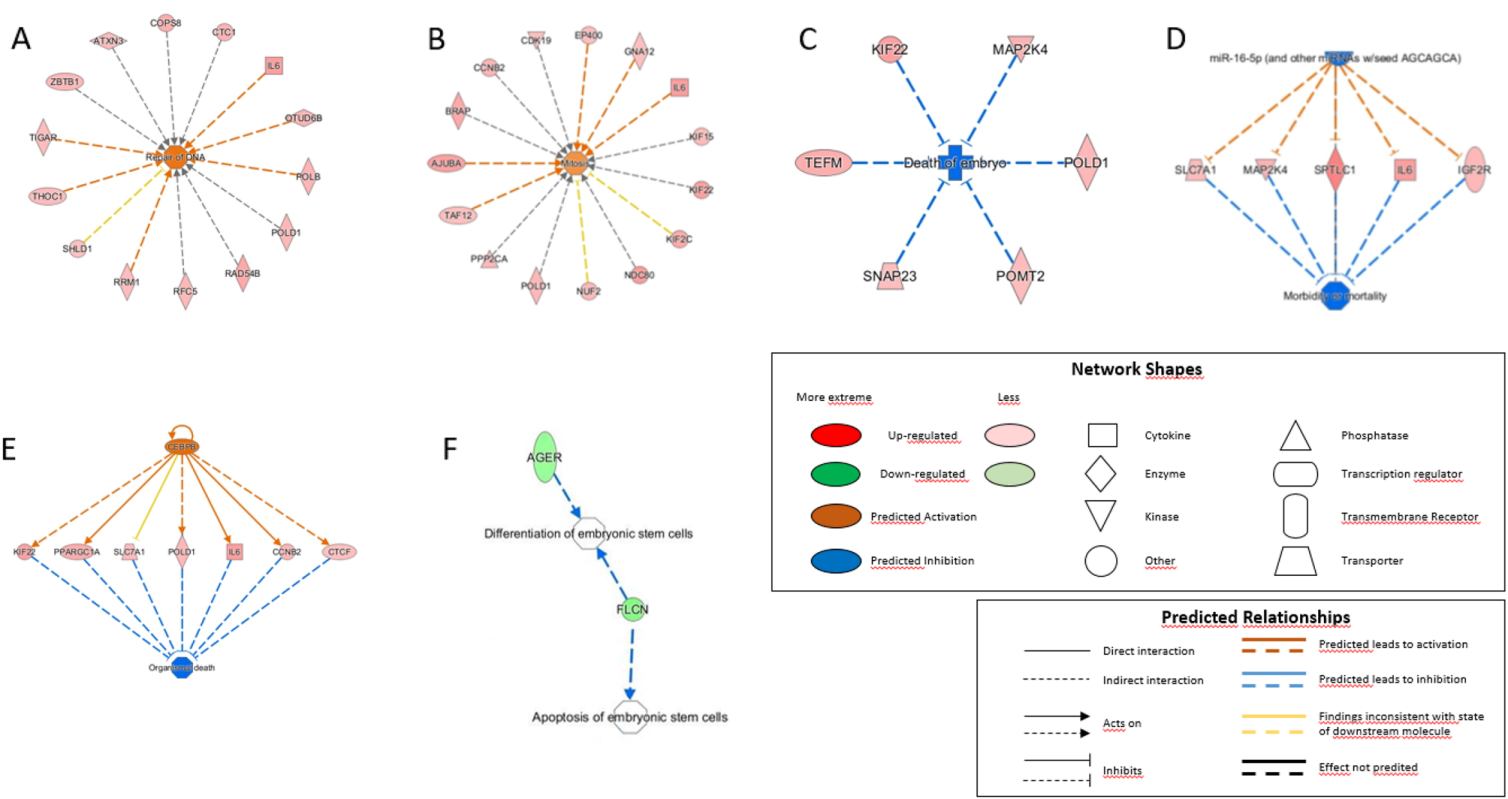
Functional annotation of the differentially expressed genes using the Ingenuity Pathway Analysis.

The IPA system was used for the bioinformatic analysis. IPA is a powerful analysis and search tool to evaluate the significance of “omics” data and to identify novel mechanistic pathways^30^. Analysis of the differentially expressed RNAs using IPA, as previously described^30^, identified the signaling and metabolic pathways, upstream regulators, molecular interaction networks, and disease and biological functions that were most likely to be perturbed by their deregulation (Table 4 and Fig. 3).

**Table 4:** Functional annotation of differentially expressed genes by Ingenuity Pathway Analysis (IPA). To investigate possible interactions of the differentially expressed RNAs, the datasets including the 663 upregulated RNAs (A, B, and C) and the 44 downregulated RNAs (D and E) in blastocysts cultured in biphasic (5%-2%) O_2_ concentration compared with monophasic (5%) O_2_ concentration were imported in the IPA Tool to identify the top signaling pathways, functions and interactions. A general overview of the pathways linked to embryo and fetal development are presented in the table with the number and the names of transcripts from the datasets that map to each pathway. For A, B, D and E, the right-tailed Fisher's exact test was used by IPA to determine whether the biological attribute was significantly enriched in the dataset (*p* ≤ 0.05 was considered significant). For C, the two highest consistency scores are presented. The consistency score is an indicator used to describe the causal consistency of the upstream regulatory factor in the network, the dataset and dense connection metric between disease and function.

A	Top molecular and cellular functions	*P* value range	# Transcripts	Transcript names
	RNA post-transcriptional modification	8.43 × 10^–3^–2.73 × 10^–4^	16	ADARB1, ATXN3, CTDP1, DDX52, GEMIN2, INTS8, INTS9, PELP1, PPARGC1A, RBPMS, SF3B6, SNRNP27, SRSF5, SYF2, THOC1, TRNT1
	Cellular assembly and organization	1.50 × 10^–2^–3.32 × 10^–4^	25	ADARB1, ATXN3, AURKC, BNIP3L, CCNB2, CTSV, DDX19B, DGUOK, DST, EHBP1, GNA12, IFT20, IL6, KIF22, KIF2C, LAMTOR1, MMS22L, NDC80, NUBPL, NUF2, PPARGC1A, PRKAA1, SMC6, SUCO, VDAC3
	DNA replication, recombination and repair	1.08 × 10^–2^–3.32 × 10^–4^	26	ADARB1, ATXN3, AURKC, CCNB2, COPS8, CTC1, DGUOK, IL6, KIF22, KIF2C, MMS22L, NDC80, NUF2, OARD1, OTUD6B, POLB, POLD1, RAD54B, RBBP5, RFC5, RRM1, SHLD1, SMC6, THOC1, TIGAR, ZBTB1
	Cell cycle	1.77 × 10^–2^–6.91 × 10^–4^	22	AJUBA, AURKC, BRAP, CCNB2, CDK19, CHKA, CTCF, EP400, GNA12, IL6, KIF15, KIF22, KIF2C, NDC80, NUF2, POLD1, PPP2CA, PRKAA1, RRM1, SMC6, TAF12, ZBTB1
	Cell death and survival	1.49 × 10^–2^–6.91 × 10^–4^	26	AKIP1, ATXN3, BAK1, BNIP3L, CHKA, CTH, DAPK1, EMC4, EP400, IL6, KIF15, MAP2K4, MINPP1, MMS22L, NDC80, NUF2, PPARGC1A, PP2CA, PRKAA1, RIPK1, SHLD1, SMC6, STK3, TFB1M, THOC1, TM2D1

	Top physiological system development and function	*P* value range	# Transcripts	Transcript names
	Organismal functions	1.08 × 10^–2^–3.32 × 10^–4^	7	CTCF, DGUOK, IL6, MAP2K4, POMT2, PPARGC1A, PRKAA1
	Digestive system development and function	1.12 × 10^–2^–3.48 × 10^–4^	10	ARV1, CTSV, DGUOK, IL6, MAP2K4, mir-597, POLB, POLD1, PPARGC1A, STK3
	Hepatic system development and function	1.12 × 10^–2^–3.48 × 10^–4^	10	ARV1, DGUOK, IGF2R, IL6, MAP2K4, mir-597, POLB, POLD1, PPARGC1A, STK3
	Organ development	1.27 × 10^–2^–3.48 × 10^–4^	11	ARV1, CTSV, DGUOK, IL6, MALT1, mir-597, POLB, POLD1, PPARGC1A, PRKAA1, STK3
	Embryonic development	1.26 × 10^–2^–3.14 × 10^–3^	11	CTCF, IL6, KIF22, MALT1, MAP2K4, POLD1, POMT2, PPARGC1A, SNAP23, TEFM, THOC1

B	Diseases or functions annotation	*p* value	# Transcripts	Transcripts in "Embryonic Development"
	Lack of embryoblast	3.14E−03	2	CTCF,THOC1
	Angiogenesis of cerebellum	1.08E−02	1	IL6
	Differentiation of Th22 cells	1.08E−02	1	IL6
	Development of anterior tibial muscle	1.08E−02	1	PPARGC1A
	Cellularity of mesenteric lymph node	1.08E−02	1	MALT1
	Cellularity of cervical lymph node	1.08E−02	1	MALT1
	Death of embryo	1.26E−02	6	KIF22,MAP2K4,POLD1,POMT2,SNAP23,TEFM

C	Disease and functions	Consistency Scores	# Transcripts	ID Regulator
	Morbidity or mortality	2.236	5	mi-R-16-5p (and other miRNAs we/seed AGCAGCA)
	Organismal death	− 3.402	7	CEBPB

D	Top molecular and cellular functions	*P* value range	# Transcripts	Transcript names
	Cellular movement	3.92 × 10^–2^–2.18 × 10^–4^	3	AGER, NEO1, SLC41A2
	Cell death and survival	3.65 × 10^–2^–9.06 × 10^–4^	9	AGER, AMBRA1, BTBD10, CABLES1, FLCN, LTBP1, NEO1, NUP160, SLC41A2
	Cell-to-cell signaling and interaction	3.57 × 10^–2^–9.06 × 10^–4^	4	AGER, AMBRA1, LTBP1, NEO1
	Cellular assembly and organization	3.04 × 10^–2^–1.81 × 10^–3^	5	AGER, FLCN, KLHL15, NEO1, NUP160
	Cellular compromise	8.13 × 10^–3^–1.81 × 10^–3^	1	AGER

	Top physiological system development and function			
	Skeletal and muscular system development and function	4.96 × 10^–2^–2.18 × 10^–4^	5	AGER, KLF11, LTBP1, NEO1, SLC41A2
	Cardiovascular system development and function	4.78 × 10^–2^–9.06 × 10^–4^	4	AGER, KLF11, LTBP1, SLC41A2
	Nervous system development and function	3.74 × 10^–2^9.06 × 10^–4^	3	AGER, KLHL15, NEO1
	Organismal development	4.96 × 10^–2^–.06 × 10^–4^	7	AGER, CABLES1, FLCN, KLF11, LTBP1, NEO1, SLC41A2
	Tissue morphology	4.44 × 10^–2^–9.06 × 10^–4^	2	AGER, LTBP1

E	Cell death and survival	*P* value	# Transcripts	Transcript names
	Apoptosis of embryonic stem cells	3.57E−02	1	FLCN
	Organismal development	*P* value	# Transcripts	Transcript names
	Differentiation of embryonic stem cells	6.68E−03	2	AGER,FLCN

For the upregulated RNAs, the top-five “Molecular and Cellular Functions” included “RNA Post-Transcriptional Modification” (16 RNAs, p value range 8.43 × 10^–3^–2.73 × 10^–4^), “Cellular Assembly and Organization” (25 RNAs, p value range 1.50 × 10^–2^–3.32 × 10^–4^), “DNA Replication, Recombination and Repair” (26 RNAs, p value range 1.08 × 10^–2^– 3.32 × 10^–4^), “Cell Cycle” (22 RNAs, p value range 1.77 × 10^–2^–6.91 × 10^–4^), and “Cell Death and Survival” (26 RNAs, p value range 1.49 × 10^–2^–6.91 × 10^–4^) (Table 4A). In “DNA Replication, Recombination and Repair”, 14 upregulated RNAs were associated with DNA repair (*p* = 1.48 × 10^–3^), among which six (*TIGAR, THOC1, RRM1, POLB, OTUD6B,* and *IL6*) are predicted to support DNA damage repair (Fig. 3A). In “Cell Cycle”, fifteen upregulated RNAs were involved in mitosis (*p* = 1.39 × 10^–2^) among which five (*AJUBA, TAF12, IL6, GNA12* and *EP400*) are predicted to stimulate this process (Fig. 3B). Moreover, upregulated RNAs in “Physiological System Development and Function” included genes implicated in “Organ Development” (11 RNAs, p value range 1.27 × 10^–2^–3.48 × 10^–4^) and “Embryonic Development” (11 RNAs, p value range 1.26 × 10^–2^–3.14 × 10^–3^) (Table 4A). “Embryonic development” included *CTCF* (CCCTC-Binding Factor), *IL6* (interleukin 6), *KIF22* (kinesin family member 22), *MALT1* (MALT1 paracaspase), *MAP2K4* (mitogen-activated protein kinase 4), *POLD1* (DNA polymerase delta 1, catalytic subunit), *POMT2* (protein O-mannosyltransferase 2), *PPARGC1A* (PPARG coactivator 1 alpha), *SNAP23* (synaptosomal-associated protein 23), *TEFM* (transcription elongation factor, mitochondrial), and *THOC1* (THO complex 1) (Table 4A). *CTCF* and *THOC1* were associated with lack of embryoblast (Table 4B). *KIF22, MAP2K4, POLD1, POMT2, SNAP23*, and *TEFM* are considered to participate in embryonic death inhibition (Table 4B and Fig. 3C). The two “Top Regulator Effect Networks” of the upregulated RNAs were initiated by *miR-16-5p* (Table 4C and Fig. 3D) and *CEBPB* (Table 4C and Fig. 3E). Inhibition of mirR-16-5p was predicted to stimulate *SLC7A1, MAP2K4, SPTLC1, IL6*, and *IGF2R*, leading to morbidity or mortality inhibition (Fig. 3D). *CEBPB* upregulation might increase the expression of *KIFF22, PPARGC1A, POLD1, IL6, CCNB2*, and *CTCF*, contributing to the inhibition of organismal death (Fig. 3E).

The top five “Molecular and Cellular Functions” of downregulated RNAs included “Cellular Movement” (3 RNAs, *p* value range 3.92 × 10^–2^–2.18 × 10^–4^), “Cell Death and Survival” (9 RNAs, *p* value range 3.65 × 10^–2^- 9.06 × 10^–4^), “Cell-to-cell Signaling and Interaction” (4 RNAs, *p* value range 3.57 × 10^–2^–9.06 × 10^–4^), “Cellular Assembly and Organization” (5 RNAs, *p* value range 3.04 × 10^–2^–1.81 × 10^–3^), and “Cellular Compromise” (1 RNAs, *p* value range 8.13 × 10^–3^–1.81 × 10^–3^) (Table 4D). In “Cell Death and Survival”, *FLCN* (folliculin) is predicted to inhibit embryonic stem cell apoptosis (*p* = 3.57 × 10^–2^). In “Organismal Development”, *AGER* and *FLCN* should maintain embryonic stem cell pluripotency by inhibiting their differentiation (*p* = 6.68 × 10^–3^) (Table 4E and Fig. 3F).

## Discussion

Our results show that embryo culture in biphasic (5–2%) O_2_ concentration is associated with a significant increase in total and usable blastocyst rates and also LBR compared with culture in monophasic (5%) O_2_ concentration. This suggest that mimicking the physiological O_2_ concentration during extended human embryo culture could increase IVF outcomes and improve the management of infertile couples. Moreover, O_2_ concentration reduction from 5 to 2% led to a significant differential gene expression in human blastocysts, corroborating the hypothesis that controlling the embryo microenvironment during in vitro culture is important to optimize blastocyst development and implantation potential. Our study is the first to provide informative data on the impact of embryo culture in biphasic (5–2%) O_2_ concentration on cumulative IVF outcomes and to identify several regulatory signaling pathways that might explain the obtained results.

In this study, the usable blastocyst rates was higher when embryos were cultured in biphasic (5–2%) O_2_ concentration, thus increasing the number of usable blastocysts to transfer per cycle and improving IVF outcomes^31^. Moreover, the higher percentage of embryo transfers associated with at least one live birth also stresses the positive impact of the biphasic (5–2%) O_2_ concentration strategy on the blastocyst implantation competence. Our results indicate that the biphasic (5–2%) O_2_ concentration strategy increases not only the number of usable blastocysts obtained in one IVF cycle, but also improves their implantation potential. In the transcriptome study, the results of the functional annotation of the differentially expressed genes by IPA could give some clues on the underlying regulatory mechanisms. Indeed, we observed that the biphasic (5–2%) O_2_ concentration strategy influenced different key pathways involved in embryo development and implantation potential acquisition (e.g. Cellular Assembly and Organization, DNA Replication, Recombination and Repair, Cell Cycle, Cell Death and Survival, Organismal Functions, Organ Development, Embryonic development and Organ Morphology). Moreover, the biphasic (5–2%) O_2_ concentration strategy modulated the expression of various genes that promote DNA repair, cell proliferation and embryonic stem cell pluripotency, and that inhibit embryo death and apoptosis. Although the transcriptome analysis was performed on a limited number of embryos (i.e. 12 in total, 6 for each group), it suggests that the biphasic (5–2%) O_2_ concentration strategy increases the expression of key transcripts supporting embryo growth and implantation potential. More experiments are now required to confirm these preliminary data and to determine whether these molecular candidates are the cause or the consequence of the increased blastocyst rate and LBR.

Recently, three studies compared the impact of biphasic (5–2%) versus monophasic (5%) O_2_ concentration strategies during human preimplantation embryo culture^10,12,16^. The significant increase in the total and usable blastocyst rates reported in our study is in agreement with two of these studies^12,16^, whereas the third one did not find any difference^10^. These three previous studies had major limitations, particularly they did not report key data (e.g. women’s age or smoking status). Indeed, O_2_ concentration impact on embryo development could vary in function of the women’s clinical and biological parameters. For example, increasing age is associated with lower O_2_ consumption by morulae^18^ and with downregulation of pro-implantation transcript expression in blastocysts^17^. Altogether, these data suggest that embryos generated using oocytes from older women could display lower adaptive abilities, and be more sensitive to oxidative damages (particularly Reactive Oxygen Species, ROS) than embryos from younger women. This might also explain the result discrepancies in these published studies. In humans, high ROS levels in culture medium have been associated with lower fertilization and cleavage rates, higher embryonic fragmentation, lower blastocyst rate, and lower pregnancy rates in IVF cycles^32^. Yang et al. observed a positive correlation between H_2_O_2_ concentration and DNA fragmentation level of unfertilized human oocytes and embryos^33^. Moreover, ROS increase the mitochondrial DNA mutation rate, leading to the arrest of in vitro embryo development^34^. Oxidative damage can cause aggregation of cytoskeleton components and endoplasmic reticulum accumulation, resulting in the formation of inclusion bodies^35^, embryo fragmentation, or development arrest^36^. One approach to limit oxidative damage is to prevent the generation of excessive free radicals by reducing O_2_ concentration during in vitro embryo culture. It could be hypothesized that the biphasic (5–2%) O_2_ concentration strategy is associated with lower ROS levels, thus reducing embryo oxidative damage. More experiments are required to confirm this hypothesis.

Our study has five major strengths. First, we evaluated the same couples in both study arms for the primary objective, which minimizes the impact of patient-dependent variables, such as genetics and lifestyle. Moreover, many clinical and biological parameters (e.g. body morphology and uterine cavity) should have remained stable. Second, we studied the impact of O_2_ concentration on 1060 D3 embryos, which is a respectable number compared to the studies by Kaser et al. (n = 176 embryos,^16^) and by Morin et al. (n = 670 D3 embryos,^12^). De Munck et al.^10^ assessed the largest embryo cohort to date (n = 1955 embryos), but they split the cohort into two studies with different protocols (n = 811 for study I and n = 1144 for study II), thus reducing the statistical power of the cohort, which becomes similar to ours^10^. Third, we used the same materials (culture media, culture dishes, and incubators) throughout the study period from June 2014 and March 2019, thus avoiding confounding factors due to different consumable brands and protocols. For example, it has been reported that the incubator type can affect the O_2_ concentration recovery rates inside the incubator (i.e. when opening/closing the incubator door), and this could influence the embryo quality, especially at the blastocyst stage^37^. Moreover, all culture dishes with medium were systematically incubated the day before use. This is considered as the optimal procedure because few hours are needed to equilibrate the O_2_ concentration in the droplets between the medium and the incubator atmosphere^10^. Fourth, we evaluated the cumulative LBR, which is the best indicator of the IVF cycle success^25^. The “one-and-done” approach (i.e. to obtain all the desired children by several fresh and frozen embryo transfers generated in only one IVF cycle) is the current ultimate goal of both patients and physicians^38^. Here, we demonstrated that the shift to a biphasic (5–2%) O_2_ concentration strategy during preimplantation embryo culture increases the chance of having a family following only one ovarian stimulation cycle and puncture. Fifth, we combined the clinical study with a transcriptome analysis of human IVF embryos the results of which allowed proposing hypotheses on the regulatory mechanisms underlying the clinical results.

Our study is also associated with several limitations. First, our study had a retrospective design. We compared embryos of the same couples, but cultured at different times. Therefore, we cannot definitely exclude the influence of confounding factors (e.g. replacement of some consumables used for embryo culture; heterogeneous quality of different culture medium batches; acquisition of technical experience) on embryo development and IVF outcomes. However, all equipment (workflows, micromanipulators, and more importantly incubators) as well as IVF/embryo culture protocols remained identical throughout the study period. Moreover, the women’s age and IVF cycle rank were increased in the biphasic (5–2%) O_2_ concentration group, which is a logical consequence of the study design. Indeed, the same couples were enrolled for two successive IVF cycles, and embryo culture in biphasic (5–2%) O_2_ concentration was always done in the subsequent cycle. However, the increasing maternal age and IVF cycle rank are expected to have negative impacts on embryo development and LBR^39–42^, and therefore cannot explain the higher blastocyst rates in the biphasic (5–2%) O_2_ concentration group. Therefore, this limitation should not question the interest of our results, and our study still provides some knowledge on the subject. Second, the use of morphological/kinetic parameters to evaluate the blastocyst rates and to select embryo to transfer and cryopreserve is associated with a limited repeatability/reproducibility, implying that the result might be different in other clinics. Moreover, the definition of “usable blastocyst” is highly variable among IVF clinics, as illustrated by the three different definitions used in the previous studies (“> 4CC”^12^, “≥ 1A with no C inner cell mass with concurrent C trophectoderm”^16^, or “from fully compacted embryos to hatching blastocysts with a visible trophectoderm and inner cell mass”^10^. This heterogeneity could limit the extrapolation of our results. Third, there were still some unused cryopreserved embryos (n = 38 from 15 couples) at the end of our study. However, all cryopreserved embryos belonged to couples with at least one live birth in the biphasic (5–2%) O_2_ concentration group, whereas all cryopreserved embryos were used in the monophasic (5%) O_2_ concentration group. Hence, the calculation of cumulative LBR is complete. The future use of cryopreserved embryos can only lead to a maintenance or an improvement of the number of live newborns per cycle in the biphasic (5–2%) O_2_ concentration group, which suggests that this limitation does not threaten the relevance of our research.

To evaluate the value of implementing dynamic (5–2%) O_2_ strategy in IVF laboratories, a multicenter randomized control trial is now required. Embryo development and the clinical endpoints should be assessed using standardized definitions to optimize the study external validity. Moreover, it could be particularly interesting to measure ROS concentration and mitochondrial activity in embryos cultured in monophasic (5%) and biphasic (5–2%) O_2_ concentration, as well as the associated epigenetic changes.

## Conclusion

Our results suggest that the biphasic (5–2%) O_2_ concentration strategy is associated with significantly improved IVF outcomes (higher total and usable blastocyst rates and increased cumulative LBR) compared with the monophasic (5%) O_2_ concentration strategy. The wide implementation of the biphasic (5–2%) O_2_ concentration strategy for preimplantation embryo culture in IVF centers could increase the “take home baby rate”, improving IVF cost-effectiveness and the management of infertile couples. The characterization of the molecular mechanisms involved in the improvement of human embryo development and implantation potential when using the biphasic (5–2%) O_2_ concentration strategy could lead to the identification of new therapeutic molecules. Randomized control trials are now needed to robustly assess these interesting data.

## Supplementary Information

Supplementary Table S1.
