# Author Correction: Biphasic (5–2%) oxygen concentration strategy significantly improves the usable blastocyst and cumulative live birth rates in in vitro fertilization

**DOI:** 10.1038/s41598-022-11988-x

**Published:** 2022-05-09

**Authors:** Sophie Brouillet, Chloé Baron, Fatima Barry, Aneta Andreeva, Delphine Haouzi, Anna Gala, Alice Ferrières-Hoa, Vanessa Loup, Tal Anahory, Noémie Ranisavljevic, Laura Gaspari, Samir Hamamah

**Affiliations:** 1 INSERM 1203, Développement Embryonnaire Fertilité Environnement, Univ Montpellier, Montpellier, France; 2 CHU Montpellier, Département de Biologie de la Reproduction, Biologie de la Reproduction/DPI et CECOS, Univ Montpellier, Montpellier, France; 3 CHU Montpellier, Unité d’Endocrinologie-Gynécologie Pédiatrique, Service de Pédiatrie, Univ Montpellier, Montpellier, France

Correction to: *Scientific Reports* 10.1038/s41598-021-01782-6, published online 17 November 2021

The original version of this Article contained an error in the spelling of the author Tal Anahory which was incorrectly given as Tal Anahoryl. The original Article has been corrected.

